# Postoperative Complications and Factors Associated with Surgical Site Infection at Muhimbili National Hospital, Dar es Salaam, Tanzania: A cross-Sectional study

**DOI:** 10.24248/eahrj.v8i2.782

**Published:** 2024-06-26

**Authors:** Amos Mbowella, Mabula Mchembe, Godbless Massawe, Ally Mwanga, Irene. A. Msoffe

**Affiliations:** a Muhimbili University of Health and Allied Sciences, Dar es Salaam, Tanzania; b Muhimbili National Hospital, Dar es Salaam, Tanzania

## Abstract

**Background:**

Postoperative complications are a leading cause of morbidity and mortality to surgical patients. Different complications are encountered in clinical practice, however surgical site infection (SSI) appears to be the most common. To date, limited published information is known pertaining to the patterns of postoperative complications and factors associated with SSI among patients operated on in other hospitals in Tanzania and referred to Muhimbili national hospital for further management. Therefore, the purpose of this study was to identify patterns of postoperative complications and factors associated with SSI among the study participants.

**Methods:**

This was a hospital based cross-sectional study conducted at Muhimbili national hospital from August 2022 to January 2023, which included 181 patients. Obtained data was analysed using frequency tables, Pearson Chi-squared test and binary logistic regression model, at a level of significance of <0.05.

**Results:**

One hundred eighty one (181) patients were included in this study, of whom 113(62.4%) were females; median age was 39 years. Cigarette smokers and alcohol consumers were 42(23.2%) and 90(49.7%) respectively. Diabetic patients were 8(4.4%), 35(19.3%) were HIV positive and 52(28.7%) had hypertension. Patients whose diagnosis was related to general surgery accounted for 50.2%, whereas 30.9% were obstetric cases. Patients who had undergone Caesarean section were 45(24.9%), whereas 20(11%) patients had undergone total abdominal hysterectomy. Bowel resection and primary anastomosis included 16(8.8%) patients and perforated peptic ulcer repair 8(4.4%) patients. Patients with infected peritoneal collection were 34(18.8%), postpartum haemorrhage 18(9.9%) and bowel perforation 10(5.5%). Patients who had undergone relaparotomy as part of treatment were 70(38.7%), whereas 30(16.6%) patients with SSIs were treated by serial wound dressing and 26(14.4%) patients were admitted and nursed in intensive care unit. None of the factors was found to have a statistically significant association with SSI.

**Conclusions:**

There is a large proportion of patients suffering from postoperative complications after gastroenterology and obstetric surgeries; and of all complications encountered in this study; SSI was the most common contributing 52%, followed by gastrointestinal complications at 31%. Despite the fact that multiple factors were associated with SSI, none of them was found to be statistically significant.

## BACKGROUND

Postoperative complications are pathological outcomes that occur after surgery characterized by physical and physiological deterioration thus hindering patients' well-being; such events increase the chance of mortality significantly.^1^ A wide range of postoperative complications is encountered, however SSIs appear to be more common; and it is classified into three groups by Centre for Disease Control and prevention.

Superficial incisional SSI is defined as that infection which occurs within 30 days after any operative procedure and involve only the skin and subcutaneous tissue of incision. The patient must also have one of the following features and conditions: purulent drainage from incision, organisms identified from an aseptically obtained specimen, superficial incision that is deliberately opened by a surgeon or other designee and culture or non–culture-based testing is not performed, and at least one of the following signs or symptoms must be present, pain or tenderness, localized swelling, erythema, or heat.^2^

Deep incisional SSI occurs within 30 or 90 days after the operative procedure and involve deep soft tissues of the incision (fascial and muscle layers).

The patient must also have at least one of the following, purulent drainage from the deep incision, a deep incision that spontaneously dehisces or is deliberately opened or aspirated by a surgeon, attending physician, or other designee, and the organism is identified by a culture or non–culture based microbiologic testing method. The patient must also have one of the following: fever, localized pain, or tenderness, an abscess or other evidence of infection involving the deep incision that is detected on gross anatomic or histopathologic examination or imaging test.^2^

Organ/space SSI, occurs within 30 or 90 days after the operative procedure and involves any part of the body deeper than the fascial/muscle layers that is opened or manipulated during the operative procedure and the patient has one of the following, purulent drainage from a drain that is placed into the organ/space, organisms are identified from an aseptically obtained fluid or tissue in the organ/space by a culture or non–culture-based microbiologic testing method. An abscess or other evidence of infection involving the organ/space that is detected on gross anatomic or histopathologic examination or imaging test.^2^

Studies conducted in Eastern Ethiopia and America revealed that cigarette smoking^1^ and alcohol consumption^3^ were associated with SSI, others identified comorbidities such as HIV-AID^4,^ diabetes mellitus and hypertension^5^ to have a significant association with SSI. Regarding urgency of the procedures, studies have revealed that among those who succumb SSI had undergone surgery on emergency basis^(6, 7)^

There was no published report to account for surgical complications and risk factors for SSI among surgical patients in Tanzania. Therefore, this study was intended to assess patterns of postoperative complications and factors associated with SSI among patients operated in other hospitals in Tanzania and referred to Muhimbili national hospital (a tertiary referral hospital) for further management.

## METHOD

### Study Area and Period

The study was conducted in the United Republic of Tanzania at Muhimbili hospital from 1^st^ of August 2022 to 30^th^ of January 2023. Muhimbili hospital is a national referral and a teaching hospital located in the city of Dar es Salaam.

### Study Design and Population

This was a hospital based cross-sectional study design conducted among 181 patients. The study population included all patients who had undergone surgery in any hospital in Tanzania and later referred to Muhimbili National Hospital for further management after developing postoperative complication(s).

### Inclusion and Exclusion Criteria

All patients aged 1 year and above who had undergone surgery in any Tanzanian hospital, developed complication(s) and finally referred to Muhimbili national hospital for further management. However, those that had incurred surgical complications following surgery from other specialties apart from general surgery, obstetrics and gynecology, urology and pediatric surgery were excluded from the study.

### Sample Size Estimation and Technique of Sampling

A sample size of 177 patients was estimated using a formula for estimating population proportion^8^ and study participants were obtained through convenience sampling technique.

### Formula for Sample Size Estimation

**{N = Z^2^ P(100-P)/ε^2^}**

where, N=Estimated Sample size, Z=standard normal deviate of 1.96, P=Prevalence of postoperative complications of 12.5%, obtained from a comparable study conducted in eastern Ethiopia^1^, ε=Margin of error of 5% (i.e. less than half of the prevalence). Plugging the values into the formula, **{1.96^2^ × 12.5 % (100% - 12.5 %)/5 %^2^} = 168,** adjusting for non-response rate of 5%, assuming 95% response rate **(1/0.95)×168 = 177** patients.

### Study Variables

Gender, age, nature of operation, level of education, cigarette smoking, alcohol consumption, diabetes mellitus, hypertension and HIV status were the independent variables while SSI was the dependent variable.

### Data Collection

Data was collected from 181 patients with postoperative complications referred to Muhimbili national hospital. These were admitted either through emergency department, maternity block or through out patient department, respective wards including general surgery, maternity, gynecology, urology, pediatric surgery, surgical and maternity ICU were visited on daily basis aim was identify newly admitted patients with the required inclusion criteria's, whenever patients were encountered desired information was collected from them and sometimes primary care takers especially for critically ill patients. After establishing rapport, consent was sought from every patient followed by in-depth interview using a well-structured questionnaire which aimed at capturing information's pertaining sociodemographic and clinical characteristics of patients. General and local examinations were also done to identify complications such as SSIs. Referral forms were then reviewed and required information was recorded, this included indication of surgery, type of surgery done, type of complication incurred and reason for referral. Thereafter patient case notes and files were reviewed to obtain information's about definitive treatment for which the patient has received. Obtained data was recorded and stored on Microsoft excel.

### Data Analysis

Collected data was entered into Statical Package for Social Sciences version 23, data proofreading and cleaning was done during data entrance. Frequency tables describing patients with post operative complication(s) were formulated. Odds ratio was used to establish association between factors predicting SSI. Univariate analysis was done using Pearson Chi-squared test followed by multivariable analysis with binary logistic regression model to control for confounders and a *P* value less than 0.05 was considered statistically significant.

### Ethical Considerations

Ethical clearance to conduct the study was obtained from Muhimbili University of Health and Allied Sciences Senate research and publications committee, and a code of ethical authorization was “DA.282/298/01.C/1272”. Muhimbili National Hospital granted permission with reference number “MNH/TRCU/perm/2022/106” to collect data.

## RESULTS

181(100%) patients were included in the study. 113(62.4%) were females, with male to female ratio of 1:1.66 and median age of 39 years.93(51.4%) patients had primary level of education. Cigarette smokers and alcohol consumers were 42(23.2%) and 90(49.7%) respectively. Some study participants had comorbidities, 8(4.4%) were diabetic, 35(19.3%) were HIV positive and 52(28.7%) had hypertension. (Table 1)

**Table 1.** Sociodemographic and Clinical Characteristics of the Study Participants (N=181)

Variable	Frequency (n)	Percentage (%)
Age (years)		
≤ 18	11	6.1
>18	170	93.9
Sex		
Male	68	37.6
Female	113	62.4
Occupation		
Employed	24	13.3
Self employed	104	57.5
None	53	29.3
Level of education		
≤primary	93	51.4
>primary	71	39.2
None	17	9.4
Cigarette smoking		
Smokers	42	23.2
Non smokers	139	76.8
Alcohol		
Alcoholic	90	49.7
Non alcoholic	91	50.3
Diabetes Mellitus		
Yes	8	4.4
No	173	95.6
Hypertension		
Yes	52	28.7
No	129	71.3
HIV-AIDS		
Yes	35	19.3
No	146	80.7
Nature of operation		
Emergency	101	55.8
Elective	80	44.2

Cases related to general surgery were common, contributing 91(50.5%) patients. Intestinal obstruction accounted for most cases in this group with 23(12.7%) cases, followed by perforated peptic ulcers 8(4.4%) cases. Other diagnosis were appendicular abscess, visceral injuries, diabetic foot ulcers, hernias, peritonitis and others. 57(31.6%) patients had preoperative diagnosis related to obstetric conditions with obstructed labor being most common contributing 16(8.8%) patients, different causes of obstruction were identified ranging from fetal malpresentation, big baby, small pelvis and others, (Table 2). 101(55.8%) patients were operated on emergency basis, 64(33%) patients underwent laparotomy and 45(24.9%) patients underwent caesarian section, due to different indications. (Table 3).

**Table 2.** Patients' Diagnosis Prior to Index Surgery (N=181)

Preoperative Diagnosis	Frequency (n)	Percentage (%)
General surgery		
Intestinal obstruction	23	12.7
Perforated peptic ulcer	8	4.4
Intra-abdominal tumor	7	3.9
Appendicular abscess	7	3.9
Visceral injury	7	3.9
Diabetic foot ulcer	6	3.3
Hernia	4	2.2
Peritonitis	3	1.7
Breast lump	3	1.7
Obstructive jaundice (HOP)	3	1.7
Soft tissue sarcoma	3	1.7
Hemorrhoids	2	1.1
Fistula in ano	2	1.1
Soft tissue injury	2	1.1
Hemothorax	2	1.1
Other diagnosis	9	5
Obstetrics		
Obstructed labour	16	8.8
Pre-eclampsia	10	5.5
Uterine rupture	9	5.0
Prolonged labour	5	2.8
Fetal malpresentation	5	2.8
Uterine atony	4	2.2
Previous scar	3	1.7
Fetal distress	2	1.1
Big baby	2	1.1
IUFD	1	0.6
Gynecology		
Molar pregnancy	6	3.3
Ovarian cancer	4	2.2
Uterine cancer	2	1.1
Ovarian cyst	1	0.6
Uterine myoma	1	0.6
Ectopic pregnancy	1	0.6
Incomplete abortion	1	0.6
Dysfunction uterine bleeding	1	0.6
Urology		
BPH	7	3.9
Bladder cancer	1	0.6
Bladder diverticulum	1	0.6
Hydrocele	1	0.6
Pediatrics		
Hernia	6	3.3
Total	181	100

**Table 3.** Surgical Procedures Done in Primary Hospitals (N= 181)

Clinical variable	Frequency (n)	Percentage (%)
Index surgery		
General surgery		
Bowel resection & primary anastomosis	16	8.8
Perforated Peptic ulcer repair	8	4.4
Laparotomy for appendicular abscess drainage	7	3.9
Lower limb amputation	6	3.3
Tube thoracostomy	6	3.3
De-functioning colostomy	5	2.8
Mesenteric tumor open biopsy	5	2.8
Adhesionalysis	4	2.2
Laparotomy for peritoneal lavage	4	2.2
Hernioplasty	4	2.2
Soft tissue sarcoma wide local excision	4	2.2
Breast lumpectomy	3	1.7
Roux-en-Y choledochojejunostomy	3	1.7
Hemorrhoidectomy	2	1.1
Fistulotomy (Fistula in ano)	2	1.1
Debridement of Soft tissue injury (upper limbs)	2	1.1
Gastrectomy	1	0.6
Splenectomy	1	0.6
Liver laceration repair	1	0.6
Duodenal tear repair	1	0.6
Gastric feeding tube	1	0.6
Open Liver biopsy	1	0.6
Liver abscess drainage	1	0.6
Varicose vein stripping	1	0.6
Skin grafting	1	0.6
Fasciotomy	1	0.6
Obstetrics and Gynecology		
Caesarian section	45	24.9
Total abdominal hysterectomy (TAH)	20	11.0
Salpingo-ophorectomy	5	2.8
Uterine curettage	2	1.1
Ovarian cystectomy	1	0.6
Urology		
Open prostatectomy	7	3.9
Hydrocelectomy	1	0.6
Urinary bladder diverticulectomy	1	0.6
Cystectomy and urine diversion	1	0.6
Urinary bladder repair post injury	1	0.6
Pediatrics		
Herniotomy	6	3.3
Total	181	100

Of all complications encountered, infectious complications occurred most.38 (21%) patients had organ/space SSI of which 34(18.8%) of them had infected peritoneal collection and 4(2.2%) had thoracic empyema.31 (17.1%) patients had deep incisional SSI and 25(13.8%) had superficial incisional SSI. (Table 4)

**Table 4.** Postoperative Complications that Occurred After Being Operated on in Primary Hospital (N= 219)

Clinical variable	Frequency (n)	Percentage (%)
Postoperative complications		
Gastrointestinal		
Iatrogenic bowel perforation	10	5.5
Intestinal obstruction	9	5.0
Enterocutaneous fistula	8	4.4
Abdominal wall hernia recurrence	7	3.9
Burst abdomen with bowel evisceration	5	2.8
Colostomy fecal impaction	2	1.1
Anal stenosis post hemorrhoidectomy	2	1.1
Abdominal wall tumor recurrence	2	1.1
Colostomy retraction	1	0.6
Peri-colostomy bleeding	1	0.6
Gastric outlet obstruction	1	0.6
Choledochojejunostomy stricture	1	0.6
Gastrojejunostomy stricture	1	0.6
Others	6	3.3
Cardiovascular & hematological		
Postpartum hemorrhage	18	9.9
Hemoperitoneum	7	3.9
Severe anemia	5	2.8
Hemorrhagic shock	4	2.2
Septic shock	3	1.7
Upper limb vascular injury	2	1.1
Cardiac arrest	2	1.1
Postpartum cardiomyopathy	1	0.6
Genitourinary system		
Acute kidney injury	9	5.0
Ureteric injury	6	3.3
Urethral bleeding post open prostatectomy	3	1.7
Vesical vagina fistula	2	1.1
Uterus perforation post curettage	2	1.1
Gangrenous ovaries	1	0.6
Others	2	1.1
Infectious complications		
Organ/space SSI		
Infected peritoneal collection	34	18.8
Thoracic empyema	4	2.2
Deep incisional SSI	31	17.1
Superficial incisional SSI	25	13.8
Respiratory system		
Acute respiratory distress syndrome	2	1.1

At Muhimbili National Hospital (MNH), patients were treated depending on the complication, however most patients underwent re-do laparotomy for different interventions.16(8.8%) patients underwent laparotomy for lavage and defunctioning ileostomy meanwhile 13(7.2%) underwent laparotomy for lavage and defunctioning colostomy, 11(6.1%) underwent laparotomy for lavage and drain insertion, 6(3.3%) for ureteric injury repair, 5(2.8%) for total hysterectomy, 5(2.8%) for burst abdominal wall tension suture repair and others. 66(36.6%) patients were treated conservatively, 30(16.6%) of them underwent serial wound dressing, 12(6.6%) were given massive blood transfusion etc. 8(4.4%) patients were intervened surgically and nursed in ICU and 18(9.9%) patients were intervened non surgically but were admitted in ICU because of their critical conditions (Table 5).

**Table 5.** Interventions Done at Muhimbili National Hospital (N= 189)

Type of intervention done	Frequency (n)	Percentage (%)
Surgical interventions		
Redo-laparotomy		
Peritoneal lavage plus de-functioning ileostomy	16	8.8
Peritoneal lavage plus de-functioning colostomy	13	7.2
Peritoneal lavage plus drain insertion	11	6.1
Ureteric injury repair	6	3.3
Total abdominal hysterectomy	5	2.8
Burst Abdominal wall tension suture repair	5	2.8
Bowel adhesionalysis	4	2.2
Peritoneal clot evacuation plus lavage	4	2.2
Vesical vaginal fistula repair	2	1.1
Gastrojejunostomy	1	0.6
Ileo-transverse anastomosis	1	0.6
Bilateral salpingoophorectomy	1	0.6
Mesenteric tumor resection	1	0.6
Herniorrhaphy	4	2.2
Tube thoracostomy	4	2.2
Stump revision post DFU amputation	3	1.7
DFU Above knee amputation	3	1.7
Hernioplasty	3	1.7
Above elbow amputation	2	1.1
Modified radical mastectomy	2	1.1
Colostomy revision	2	1.1
Breast abscess incision and drainage	1	0.6
Anorectal abscess incision and drainage	1	0.6
Setton sutures placement	1	0.6
Abdominal wall sarcoma wide local excision	1	0.6
Non-surgical intervention		
Serial wound dressing	30	16.6
BT for PPH and other hemorrhagic conditions	12	6.6
Hemodialysis	4	2.2
Urethral irrigation	4	2.2
Serial anal dilatation	2	1.1
Colostomy enema	2	1.1
Compressional dressing for bleeding colostomy	1	0.6
Maintenance of KCL for intractable hypokalemia	1	0.6
Enema	1	0.6
Palliative Chemotherapy	1	0.6
Others	8	4.4
Intensive care unit		
Surgical intervention plus ICU	8	4.4
Non-surgical intervention plus ICU	18	9.9

Abbreviation: DFU, Diabetic Foot Ulcers; ICU, Intensive Care Unit; KCL, potassium chloride, BT, blood transfusion, PPH, postpartum hemorrhage

There were factors associated with surgical site infection among study participants, however none of them was/were found to have a statistically significant association with SSI (Table 6).

**Table 6.** Factors Associated with Surgical Site Infections Among Study Participants (N=181)

Clinical variable	Surgical Site Infection n (%)	No Surgical Site Infection n (%)	Crude P value	Adjusted P value
Age (years)				
<18	4 (36.4)	7 (63.6)	0.341	0.618
≥18	87 (51.2)	83 (48.8)		
Sex				
Male	39 (57.4)	29 (42.6)	0.14	0.326
Female	52 (46.0)	61 (54.0)		
Level of education				
>than primary	34 (47.9)	37 (52.1)	0.799	0.626
≤ primary	49 (52.7)	44 (47.3)		
None	8 (47.1)	9 (52.9)		
Cigarette smoking				
Smokers	24 (57.1)	18 (42.9)	0.427	0.605
Non smokers	63 (49.2)	65 (50.8)		
N/A	4 (36.4)	7 (63.6)		
Alcohol consumption				
Consumers	51 (56.7)	39 (43.3)	0.201	0.341
Non consumers	36 (45.0)	44 (55.0)		
N/A	4 (36.4)	7 (63.6)		
Diabetes mellitus				
Diabetic	7 (87.5)	1 (12.5)	0.031	0.076
Non Diabetic	84 (48.6)	89 (51.4)		
Hypertension				
Hypertensive	27 (51.9)	25 (48.1)	0.778	0.572
Non hypertensive	64 (49.6)	65 (50.4)		
HIV-AIDS				
Yes	20 (57.1)	15 (42.9)	0.366	0.655
No	71 (48.6)	75 (51.4)		
Nature of operation				
Emergency	49 (48.5)	52 (51.5)	0.594	0.783
Elective	42 (52.5)	(47.5)		

Abbreviations: N/A=Not Applicable; ≤ less than or equal to; > greater than; SSI=Surgical site infection.

## DISCUSSION

### Socio-demographic and clinical characteristics.

Median age of study participants was similar to studies conducted in Uganda,^9^ however in China, United Kingdom and the Netherlands the median age was higher.^10, 11, 12^ Quality health care provision and life expectancy in developed countries could explain the difference. More females were affected with surgical complications similar to studies conducted in Asia and Europe;^10, 12^ however, studies done in Ethiopia and Uganda,^1, 9^ showed male predominance. This difference is perhaps because the department of obstetrics & gynaecology contributed most patients. In this study, no factor was found to be associated with SSI. However, other studies have shown that more cigarette smokers^4^ and alcoholics^3^ developed postoperative complications as compared to nonsmokers^1^ and non-alcoholics,^3^. Cigarette smoke contains nicotine and alcohol contains formalaldehyde toxic compounds with a potential of causing immunosuppression and tissue injury, events which increase chances of surgical complications. Likewise, patients with comorbidities such as HIV-AIDS,^4^ hypertension,^4, 5^ and diabetes mellitus,^5^ are more prone to postoperative complications since they contribute to immunosuppression.

### Surgical procedures done in primary hospitals

Different surgeries were done; however, procedures related to gastroenterology were the most common. Patients underwent bowel resection and primary anastomosis, others laparotomy for abscess drainage, adhesionalysis, appendectomy, colostomy, repair of perforated bowel, graham's patch,^13,14^ hernia repair,^7^ and others. In obstetrics and gynaecology most patients underwent caesarean sections,^7,4^ and others underwent different gynaecological procedures.^15,16^ Similarities in these surgical procedures can be explained by large volume of patients with gastroenterology, obstetrics & gynaecological diseases.

### Types of complications that occurred after being operated in primary hospital

Postoperative complication is a function of multiple factors ranging from patient factors, surgeon factors and anaesthetic factors.^17^ In developing countries like Tanzania, a significant proportion of the population belong to low socioeconomic status and thus most of surgical patients are malnourished, anaemic with multiple comorbidities; ^6^ this challenge is compounded by the fact that there are few or no surgeons in some upcountry hospitals.^18^

Cardiovascular and haematological complications were also encountered in this study. Postpartum haemorrhage was the most common, followed by intraabdominal bleeding, anaemia, and haemorrhagic shock. However, in other studies, the proportions of postpartum haemorrhage^19,20^ and intraabdominal bleeding^10,14^ were significantly lower, while that of anaemia was higher.^6,9^ In Tanzania obstetric services are delivered from health centres by health attendants who lack sufficient knowledge and appropriate skills to provide quality services.^17^ Regarding anaemia, patients included in this study were only those with severe anaemia which necessitated urgent transfusion with more than four units of blood contrary to the cited studies which included all patients with mild, moderate and severe anaemia.

Two common complications related to genitourinary system noted in this study were acute renal failure and ureteric injury. In the United States of America and Japan, the incidence of renal failure was much lower.^5,10^ Postoperatively, renal failure commonly occurs because of hypovolemia caused by haemorrhage and fluid losses which require sufficient medical supplies, skilful and knowledgeable medical personnel to quickly stabilize the patient and prevent further renal injury, all of which lack in developing countries like Tanzania as compared to developed ones.^15^ This study also revealed that ureteric injuries occurred, a finding similar to a study done in northern Tanzania,^15^ but quite different from what was noted in Nigeria.^21^ A low proportion of ureteric injuries noted in this study could be due to lack of surveillance after pelvic surgeries and thus some injuries go unnoticed.^22^

Of all complications noted in this study, surgical site infection was common. In Ethiopia the incidence of SSI was significantly higher,^1^ contrary to what was observed in the United States of America.^5^ SSI being so common has been demonstrated by different studies conducted around the globe.^13,10,14^ However, in developing countries the proportion is quite high compared to developed countries which could be attributed to multiple factors such as unfriendly working environment, non-adherence to infection prevention control guidelines, patients malnutrition and multiple comorbidities.^4,5^

### Treatment done at Muhimbili National Hospital

Re-laparotomy was the leading procedure done at MNH, a finding similar to what was observed in Netherlands,^23^ whereby in India^24^, a lower proportion was noted. Procedures done after re-laparotomy included peritoneal lavage plus ileostomy or colostomy formation, peritoneal lavage plus drain insertion, exploration and abdomen closure, burst abdominal wall repair and others.^6,24,25^ The observed similarity in-terms of surgical procedures is because, worldwide surgical procedures are managed using standard guidelines thus similar conditions would be managed in a similar manner regardless of geographical differences.

### Factors associated with surgical site infections among study participants

Multivariate analysis identified none of the factors had a statistically significant association; this can be explained by presence of multiple confounding factors for SSI causation and possibly a relatively smaller sample size included.

In other studies, however, cigarette smoking has been found to be associated with SSI ^1,10,26^ this is because of toxic nicotinic compounds which are responsible for immunosuppression, cardiovascular diseases etc.^12^ Older patients were more likely to develop SSI compared to younger ones; this is true because as age increases the physiologic processes of organs and tissue progressively degenerate over time and decreases immune response.^13^ Comorbidities such as HIV-AIDS,^4^ and diabetes mellitus,^5^ were also associated with poor outcome because of immunosuppression.

Most of the patients who were operated on emergency basis succumbed infectious complications; and the explanation was that, patients were operated on in a state of shock and thus were inadequately resuscitated.^9,4^ Regarding alcohol consumption, studies have identified a significant association with SSI, alcohol is thought to cause malnutrition and immunosuppression.^12^

## CONCLUSION

There was a large proportion of patients suffering from postoperative complications following gastroenterology and obstetric surgeries. Of all the complications encountered in this study, SSI was the most common; however, none of the studied factors were found to have a statistically significant association with SSI. It is recommended to setup an action plan for mentorship and supervision to enhance knowledge and surgical skills in all upcountry hospitals in Tanzania in order to mitigate gastroenterology and obstetric surgical complications. In conjunction with this, surgeons need to know and anticipate SSI in their patients because it is the most common surgical complication. Finally, since this was a ground-breaking study in our local community, we therefore recommend studies to assess patient's outcome to be conducted in future.
